# Strain improvement of *Trichoderma harzianum* for enhanced biocontrol capacity: Strategies and prospects

**DOI:** 10.3389/fmicb.2023.1146210

**Published:** 2023-04-13

**Authors:** Ziyang Xiao, Qinqin Zhao, Wei Li, Liwei Gao, Guodong Liu

**Affiliations:** ^1^State Key Laboratory of Microbial Technology, Shandong University, Qingdao, China; ^2^Tobacco Research Institute of Chinese Academy of Agricultural Sciences, Qingdao, China; ^3^Shanghai Tobacco Group Beijing Cigarette Factory Co., Ltd., Beijing, China

**Keywords:** *Trichoderma harzianum*, biocontrol, mycoparasitism, strain improvement, fungal engineering

## Abstract

In the control of plant diseases, biocontrol has the advantages of being efficient and safe for human health and the environment. The filamentous fungus *Trichoderma harzianum* and its closely related species can inhibit the growth of many phytopathogenic fungi, and have been developed as commercial biocontrol agents for decades. In this review, we summarize studies on *T. harzianum* species complex from the perspective of strain improvement. To elevate the biocontrol ability, the production of extracellular proteins and compounds with antimicrobial or plant immunity-eliciting activities need to be enhanced. In addition, resistance to various environmental stressors should be strengthened. Engineering the gene regulatory system has the potential to modulate a variety of biological processes related to biocontrol. With the rapidly developing technologies for fungal genetic engineering, *T. harzianum* strains with increased biocontrol activities are expected to be constructed to promote the sustainable development of agriculture.

## 1. Introduction

Biotic stresses in plants are caused by diverse organisms such as fungi, bacteria, viruses, weeds, and insects (Redondo-Gómez, 2013). A recent study reassessed the figures for five staple crop losses associated with biotic stresses, showing that global crop loss estimates per crop were 21.5, 30.0, 22.6, 17.2, and 21.4% for wheat, rice, maize, potato, and soybean, respectively (Savary et al., 2019). Consequently, chemical pesticides are commonly used in agricultural systems. However, the excessive and irrational use of chemical pesticides can lead to non-target effects, potential environmental and public health risks, and the generation of resistance among pests (Jasuja, 2015; Goswami et al., 2018). In comparison, biocontrol methods employing the natural enemies of pests have the advantage of being safe with lower risks of pest resistance, resulting in them being widely used in agricultural production.

**Figure 1:**
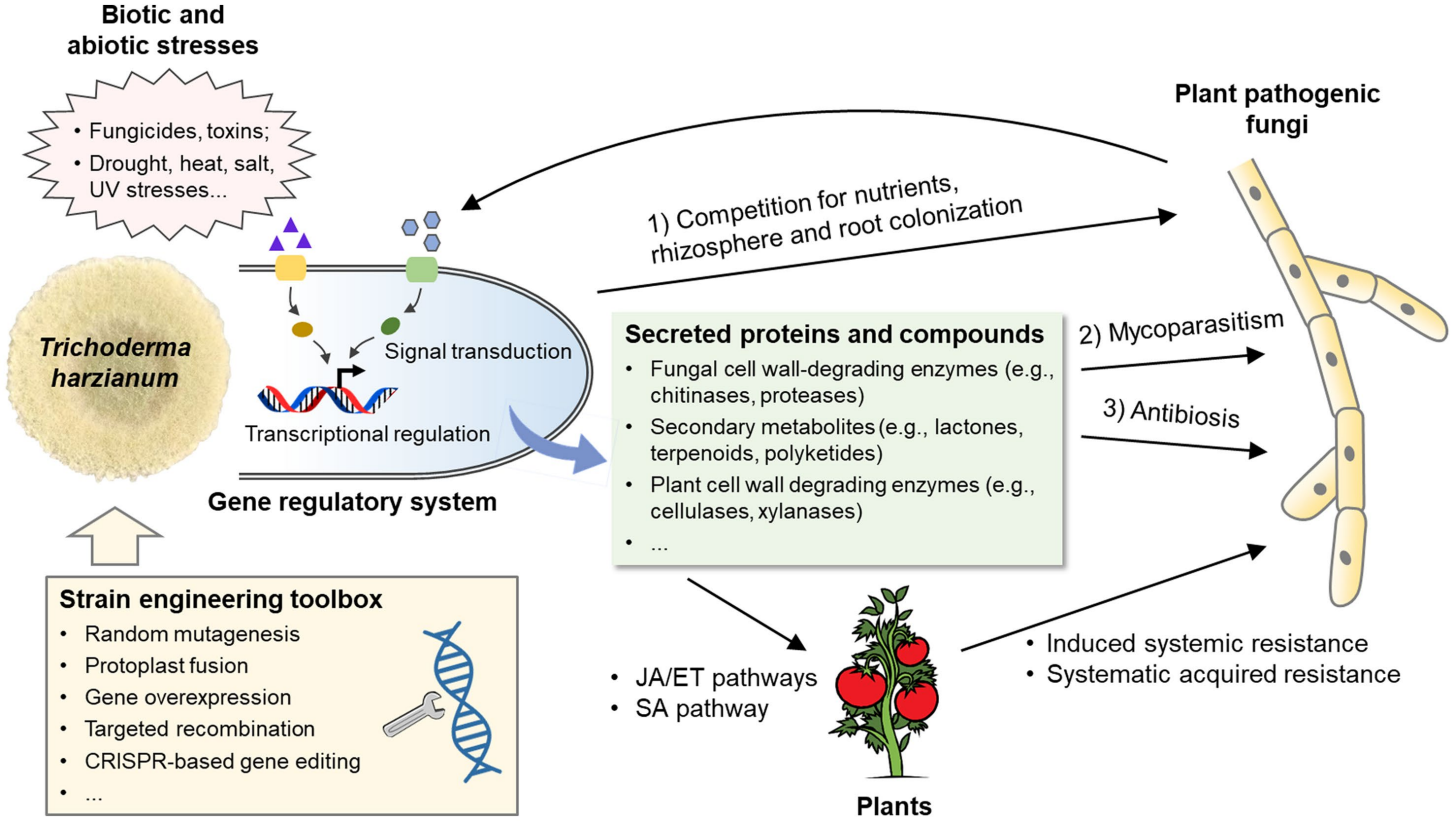
Biological processes involved in the biocontrol capacity of *Trichoderma harzianum*. Members in the *T. harzianum* species complex inhibit the growth of plant pathogenic fungi through competition, mycoparasitism and antibiosis. Meanwhile, *T. harzianum* activates defensive reactions in plants, which include induced systemic resistance and systemic acquired resistance. A set of secret proteins and secondary metabolites produced by *T. harzianum* play important roles in the above processes. In addition, *T. harzianum* is subjected to a combination of different biotic and abiotic stresses in the field. Signaling pathways and the downstream transcriptional regulation system are responsible for the regulation of responses to fungal pathogens and environmental stresses. With the genetic engineering toolbox, the biocontrol capacity of *T. harzianum* can be significantly improved. JA, jasmonic acid; ET, ethylene; SA, salicylic acid.

*Trichoderma* are well-known beneficial microorganisms in agriculture because of their ability to kill pathogenic fungi and promote plant growth (Verma et al., 2007). As biofungicides, *Trichoderma* species can inhibit the growth of many phytopathogenic fungi and oomycetes, e.g., *Fusarium solani*, *Sclerotinia sclerotiorum*, *Botrytis cinerea*, *Macrophomina phaseolina*, *Cordana musae*, *Rhizoctonia solani*, and *Pythium ultimum* (Anees et al., 2010; Samuelian, 2016; Zhang et al., 2016; Hewedy et al., 2020; Erazo et al., 2021). Inhibition is believed to involve three main mechanisms (Figure 1): (1) competition for nutrients (e.g., carbon, nitrogen, and iron) or infection spots with pathogenic fungi (Sivan, 1989; Güçlü and Özer, 2022); (2) mycoparasitism (Mukherjee et al., 2022); and (3) antibiosis through the synthesis of secondary metabolites with inhibitory or lethal effects on pathogenic fungi (El-Debaiky, 2017; Mironenka et al., 2021). In addition, *Trichoderma* species can indirectly prevent pathogen infection by inducing plant resistance responses (Harman et al., 2004; Woo et al., 2022).

*Trichoderma harzianum* is one of the most frequently used *Trichoderma* species in the management of plant diseases (Meher et al., 2020; Rush et al., 2021). It has been used for the production of more than twenty commercial biocontrol agents all over the world (Woo et al., 2014), of which eight are listed in Table 1. *T. harzianum* not only has mycoparasitic properties but also the ability to promote plant growth by adjusting the balance of hormones and acting as a biofertilizer to promote the uptake of mineral ions and carbon dioxide (Stewart and Hill, 2014; Marra et al., 2021). In the comparison of 27 *Trichoderma* species, *T. harzianum/T. afroharzianum* was found to produce the highest number of known biopesticides and plant growth-promoting compounds (Rush et al., 2021). Classical random mutagenesis (Szekeres et al., 2007; Marzano et al., 2013) and protoplast fusion (Prabavathy et al., 2006) have been successfully used to generate *T. harzianum* strains with improved performance. Along with a deeper understanding of the molecular mechanisms of biocontrol (Daguerre et al., 2014; Sood et al., 2020; Abbas et al., 2022; Chen et al., 2022), rational genetic engineering has become a feasible strategy for improving the strains of *T. harzianum* (Chen et al., 2021). Nevertheless, most of the commercial strains are reported to be wild-type isolates and no information on genetic improvement was reported. This phenomenon can be related to the restrictions and public concerns about genetically modified organisms (GMOs) (Chen et al., 2022).

**Table 1:** Selected *Trichoderma harzianum* species complex strains used for the manufacture of biocontrol products.^a^

Strain name	Product name	Product type	Manufacturer	Effectiveness
*T. afroharzianum* T-22 (T22)^b^	Trianum-P, Trianum-G	Granules containing viable spores	Koppert (Netherlands)	Inhibits the growth of *Pythium*, *Rhizoctonia*, *Fusarium*, *Botrytis* or other soil and foliar pathogenic fungi or oomycetes; promotes plant growth and uniformity
	Rootshield	Wettable powder or granules containing viable spores	BioWorks (USA)	Controls soilborne *Pythium*, *Fusarium*, *Rhizoctonia*, *Cylindrocladium*, and *Thielaviopsis*; delivers faster and stronger root development
*T. afroharzianum* G.J.S. 08–137	AkTRIvator	Powder or granules	CANNA (Netherlands)	Protects plants against soil diseases and stimulates the growth of roots and root hairs
T. *harzianum* RSTH 2222	Ecosom-TH	Wettable or soluble powder containing conidiospores	AgriLife (India)	Supression of various diseases caused by fungal pathogens; especially effective against fruit rot caused by *Botrytis* and Rhizome rot
*T. harzianum* ESALQ 1306	Trichodermil	Liquid containing viable spores	Koppert (Netherlands)^c^	Inhibits the growth of *Rhizoctonia solani*, *Sclerotinia sclerotiorum* and other fungal pathogens
*T. harzianum* T-39	Trichodex	Powder containing conidia and mycelium fragments	Makheshim-Agan (Israel)	Inhibits the growth of *Botrytis*, *Sclerotinia* and other pathogenic fungi
*T. guizhouense* CBS 134707	Promot	Powder or liquid containing viable spores	JH Biotech (USA)	Promotes beneficial microorganism populations in the root zone; stimulates root growth and promotes strong root system; induces resistance of plants

^a^Data were collected from the Bio-Pesticides DataBase (http://sitem.herts.ac.uk/aeru/bpdb/atoz.htm), Woo et al. (2014), Chaverri et al. (2015), and Meher et al. (2020). ^b^Originally derived from the fusion of strains T-95 and T-12. ^c^The product was registered in Brazil.

**Figure 2:**
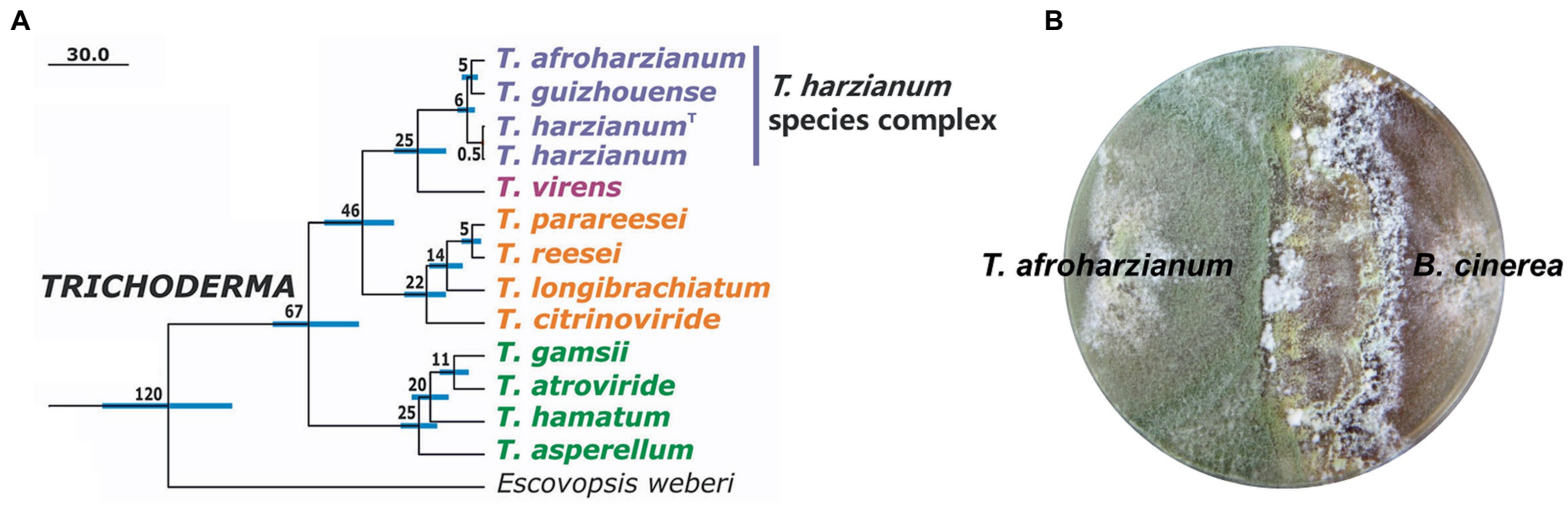
*Trichoderma harzianum* species complex for biocontrol. **(A)** Phylogenetic relationship of three species in *T. harzianum* complex and other *Trichoderma* species. The chronogram was adapted from Kubicek et al. (2019). The numbers represent chronological ages of the nodes in Mya. The NCBI GenBank accession numbers of the genomes are: *T. afroharzianum* T6776, JOKZ00000000; *T. guizhouense* NJAU 4742, LVVK00000000; *T. harzianum* CBS 226.95 (type culture, indicated with^T^), MBGI00000000; *T. harzianum* TR274, NQLC00000000; *T. virens* Gv29-8, ABDF00000000; *T. parareesei* CBS 125925, LFMI00000000; *T. reesei* QM6a, AAIL00000000; *T. longibrachiatum* ATCC 18648, MBDJ00000000; *T. citrinoviride* TUCIM 6016, MBDI00000000; *T. gamsii* T6085, JPDN00000000; *T. atroviride* IMI 206040, ABDG00000000; *T. hamatum* GD12, ANCB00000000; *T. asperellum* CBS 433.97, MBGH00000000; *Escovopsis weberi* CC031208-10, LGSR00000000. Some other species in the *T. harzianum* complex, such as *T. atrobrunneum* and *T. simmonsii*, also show good biocontrol potentials. **(B)** Overgrowth of *T. afroharzianum* T22 against plant pathogen *B. cinerea* on agar plate.

In this article, we review studies on the development of *T. harzianum* strains with enhanced biocontrol activity in laboratory level. These include the strengthening of protein and chemical effectors for biocontrol, enhancing the robustness of strains, and modulation of the gene regulatory system controlling these processes. It should be noted that with the development of systematic taxonomy in the fungal community, many previously described “*T. harzianum*” strains have been identified as other *Trichoderma* species (Mach et al., 1999; Chaverri et al., 2015; Fanelli et al., 2018; Cai and Druzhinina, 2021). For example, the strain T22, widely used as commercial biocontrol agents, was re-identified to be *T. afroharzianum* belonging to the *T. harzianum* species complex (Chaverri et al., 2015; Kubicek et al., 2019). Therefore, the review covers the research progresses in the *T. harzianum* complex (Figure 2), considering that many mechanisms for biocontrol are conserved among the members in this species complex.

## 2. Increasing the production of extracellular protein effectors

### 2.1. Fungal cell wall-degrading enzymes

Cell wall-degrading enzymes (mainly chitinases, glucanases, and proteases) play an important role in the antagonistic effect of *Trichoderma* species toward fungal pathogens. The fungal inhibitory activity of *Trichoderma* isolates was reported to be positively correlated with the production of extracellular lytic enzymes (Rai et al., 2016). As summarized below, increasing the expression of fungal cell wall-degrading enzymes is an effective strategy for enhancing the biocontrol capacity of *T. harzianum* (Table 2). Additionally, the expression of these enzymes in transgenic plants resulted in increased resistance to fungal pathogens (Distefano et al., 2008; Mercado et al., 2015).

**Table 2:** Studies on improving the biocontrol ability of *T. harzianum* strains by overexpressing fungal cell wall-degrading enzymes.

Enzyme^a^	Parental strain	Engineering strategy	Effect on inhibitory activity	Reference
ChiV (496454)	Not reported	Overexpression using CaMV35S promoter	Increase of inhibition rate against *R. solani* by 19.58%	Yang L. et al. (2011)
Chit33 (459582)	CECT 2413^b^	Overexpression using *T. reesei pki* promoter	Colony diameter of *R. solani* was about 37–67% of the control treatment	Limón et al. (1999)
Chit33 (459582)	CECT 2413^b^	Overexpression of chimeric enzyme with CBD using *T. reesei pki* promoter	Colony diameter of *R. solani* was 53–67% of the control treatment	Limón et al. (2004)
Chit42 (6140)	CECT 2413^b^	Overexpression of chimeric enzyme with CBD using *T. reesei pki* promoter	Colony diameter of *R. solani* about 65–70% of the control treatment	Limón et al. (2004)
Chit42 (6140)	ABRIICC T8-7MK	Overexpression of chimeric enzyme with ChBD using *T. reesei pki* promoter	85 and 92% reduction in *R. solani* radial growth	Kowsari et al. (2014)
Chit36 (501286)	TM^c^	Overexpression using *T. reesei pki* promoter	Stronger inhibition of *Fusarium* and *Sclerotium rolfsii* than wild type	Viterbo et al. (2001)

^a^The corresponding protein IDs in *T. afroharzianum* T-22 (https://mycocosm.jgi.doe.gov/TriharT22_1/TriharT22_1.home.html) are shown in parentheses. ^b^Also designated as ATCC 48131/CBS 354.33, originally isolated from soil. ^c^Originally isolated from Mexican soil.

#### 2.1.1. Chitinases

Chitin is a major component of the cell wall in most fungi (Brown et al., 2020). The chitinolytic system of *Trichoderma* species includes several chitinases and β-1,4-N-acetylglucosaminidases (Ghasemi et al., 2020). The most frequently studied chitinases in *T. harzianum* are Chit42/Ech42 (García et al., 1994; Carsolio et al., 1999; Woo et al., 1999), Chit33 (Limón et al., 1999; de las Mercedes Dana et al., 2001), and Chit46 (Deng et al., 2019), which are named by their molecular mass. Purified or heterologously expressed chitinases effectively inhibit the growth of phytopathogenic fungi (Wu et al., 2013). Correspondingly, introduction of the *chit42* gene to plants increased their resistances to fungal pathogens (Lorito et al., 1998).

Interspecific and intraspecific protoplasmic fusions were reported to enhance chitinase activity and antagonistic activity in *T. harzianum* (Balasubramanian et al., 2012; Hassan, 2014). On the other hand, rational genetic engineering has also been used to improve the chitinase activity of *T. harzianum* strains. Both overexpression and enzyme engineering strategies were applied to this end. Overexpression of the *chit33* gene using a constitutive promoter resulted in an approximately 200-fold increase in extracellular chitinase activity, and the inhibitory ability against *R. solani* was effectively improved (Limón et al., 1999). Moreover, the main chitinases produced by *T. harzianum* lack a specific chitin-binding domain (ChBD), which affects their affinity for insoluble chitin in the fungal cell wall. The transformants with the overexpression of a chimeric chitinase carrying ChBD from a *T. atroviride* chitinase showed higher chitinase activities and stronger inhibition against *R. solani*, compared with those without ChBD (Kowsari et al., 2014; Eslahi et al., 2021). Similarly, the addition of cellulose binding domains (CBDs) with binding ability to the chitin surface to chitinases led to not only increased chitinase activity but also more effective inhibition against *R. solani*, *B. cinerea*, and *Phytophthora citrophthora* than the wild-type strain (Limón et al., 2004).

#### 2.1.2. Glucanases

β- and α-linked glucans are also major components of the scaffold and matrix of the fungal cell wall (Kang et al., 2018). β-1,3-exoglucanase, β-1,3-endoglucanase, and β-1,6-endoglucanase have been reported to be associated with the biological control ability of *T. harzianum* (de la Cruz et al., 1996; Cohen-Kupiec et al., 1999; de la Cruz and Llobell, 1999; Donzelli et al., 2001). After contact with *F. solani*, the expression level of β-1,3-endoglucanase in *T. harzianum* was significantly upregulated compared with that before contact (Vieira et al., 2013). Furthermore, endo-β-1,3-glucanase, cellulase (β-1,4-glucanase), and α-1,3-glucanase purified from *T. harzianum* were shown to inhibit the growth of several pathogenic fungi (Thrane et al., 1997; Ait-Lahsen et al., 2001). Although gene knockout has been used to study the function of β-1,3-endoglucanase in biocontrol (Suriani Ribeiro et al., 2019), overexpression of glucanase-encoding genes for enhanced biocontrol performance has rarely been reported. An endo-β-1,6-glucanase BGN16.2 was successfully overexpressed using the *T. reesei pki* promoter; however, its effect on biocontrol ability remains to be studied (Delgado-Jarana et al., 2000).

#### 2.1.3. Proteases

In addition to chitinases and glucanases, proteases play an important role in the degradation of fungal cell walls. A proteomic study found that an aspartic protease was highly expressed in *T. harzianum* in the presence of the cell walls of *P. ultimum* and *B. cinerea* (Suárez et al., 2005). Multiple proteases from *T. harzianum*, such as serine proteases (Yan and Qian, 2009; Liu and Yang, 2013; Fan et al., 2014) and aspartic proteases (Delgado-Jarana et al., 2002; Liu and Yang, 2007; Deng et al., 2018), showed significant inhibitory activities against pathogenic fungi. After ultraviolet light (UV) irradiation, the extracellular protease activities of some *T. harzianum* mutants were 6 to 12.5 times higher than that of the wild-type strain, and certain mutants were proven to be more effective against fungal pathogens during *in vitro* plate antagonism experiments (Szekeres et al., 2004). Overexpression of the serine protease-encoding gene *prb1* was reported to increase protease production and enhance antagonistic activity against *R. solani* (Flores et al., 1997).

### 2.2. Other extracellular proteins

In addition to fungal cell wall-degrading enzymes, *T. harzianum* produces other proteins, such as plant cell wall-degrading enzymes, L-amino acid oxidase, cerato-platanins and hydrophobins, to inhibit pathogens and/or induce plant resistances.

*Trichoderma* spp. can secrete plant cell wall-degrading enzymes as elicitors to induce plant resistance to pathogens. For example, cellulases and xylanases from *Trichoderma* have been reported to induce plant defense responses *via* the ethylene/H_2_O_2_/calcium/jasmonic acid signaling pathways (Saravanakumar et al., 2016; Guo et al., 2021). By constructing a gene-silenced mutant and investigating its effect on the transcriptome of *Arabidopsis*, *Thpg1* (encoding an endopolygalacturonase) was found to be required for active root colonization and plant defense induction by *T. harzianum* T34 (Morán-Diez et al., 2009). Finally, a swollenin from *T. guizhouense* can promote the growth of cucumber by altering the root cell wall architecture (Meng et al., 2019). According to the evolutionary analysis of genes, 41% of plant cell wall-degrading enzymes and auxiliary proteins in *Trichoderma* were obtained *via* lateral gene transfer from other classes of Ascomycota (Druzhinina et al., 2018).

Proteomic analysis revealed that the expression of L-amino acid oxidase (LAAO) was induced in media containing deactivated *B. cinerea* mycelia as the sole carbon source (Yang et al., 2009). LAAO has inhibitory effects on pathogenic bacteria and fungi. For the inhibition of *R. solani*, *T. harzianum* LAAO physically interacts with the cell wall proteins of the pathogen and triggers the mitochondria-mediated apoptosis pathway, including cytochrome c release and the activation of apoptosis factors, caspases 3 and 9 (Yang C. A. et al., 2011; Yang et al., 2012).

Cerato-platanins are small, secreted cysteine-rich proteins that act as effectors and elicitors in fungus-plant interactions. Although the cerato-platanin family protein Epl1 is not necessary for the biocontrol ability of *T. harzianum*, the absence of *epl1* was found to affect the expression level of mycoparasitic genes (Gomes et al., 2015; Gao et al., 2020). Furthermore, removal of *epl1* from *T. harzianum* not only reduced the jasmonic acid-mediated defense response in tomato, but also lost its ability to downregulate the expression of *B. cinerea* virulence genes (Gomes et al., 2017; Gao et al., 2020). Another type of surface-active small protein, hydrophobin, is also involved in interactions between *Trichoderma* and plants (Viterbo and Chet, 2006). Thhdy1, a class II hydrophobin from *T. harzianum*, acts as an elicitor to activate the expression of jasmonic acid/ethylene defense-related and brassinosteroid-associated genes that are involved in plant systemic resistance (Yu et al., 2020). Therefore, the construction of *Thhdy1*-overexpressing *T. harzianum* strains is expected to enhance their biocontrol activity.

Reactive oxygen species (ROS) act as signals to regulate diverse biological processes. The production of ROS has been suggested to be one of the mechanisms of induced systemic resistance in plants by *T. harzianum* (Lara-Ortíz et al., 2003; Zhang et al., 2017). NADPH oxidases, although not extracellular proteins, are involved in the formation of ROS and are therefore indirectly associated with the biocontrol ability of *T. harzianum*. Transformants overexpressing the NADPH oxidase-encoding gene *nox1* showed higher inhibitory activity against *P. ultimum* than the wild-type. According to the result of transcriptomic analysis, the *nox1*-overexpressing transformant had upregulated expression of genes linked to protease, cellulase, and chitinase activities in the interaction with *P. ultimum* compared to the wild-type strain (Montero-Barrientos et al., 2011).

## 3. Engineering the biosynthesis of secondary metabolites

### 3.1. Bioactive compounds produced by *Trichoderma harzianum*

**Figure 3:**
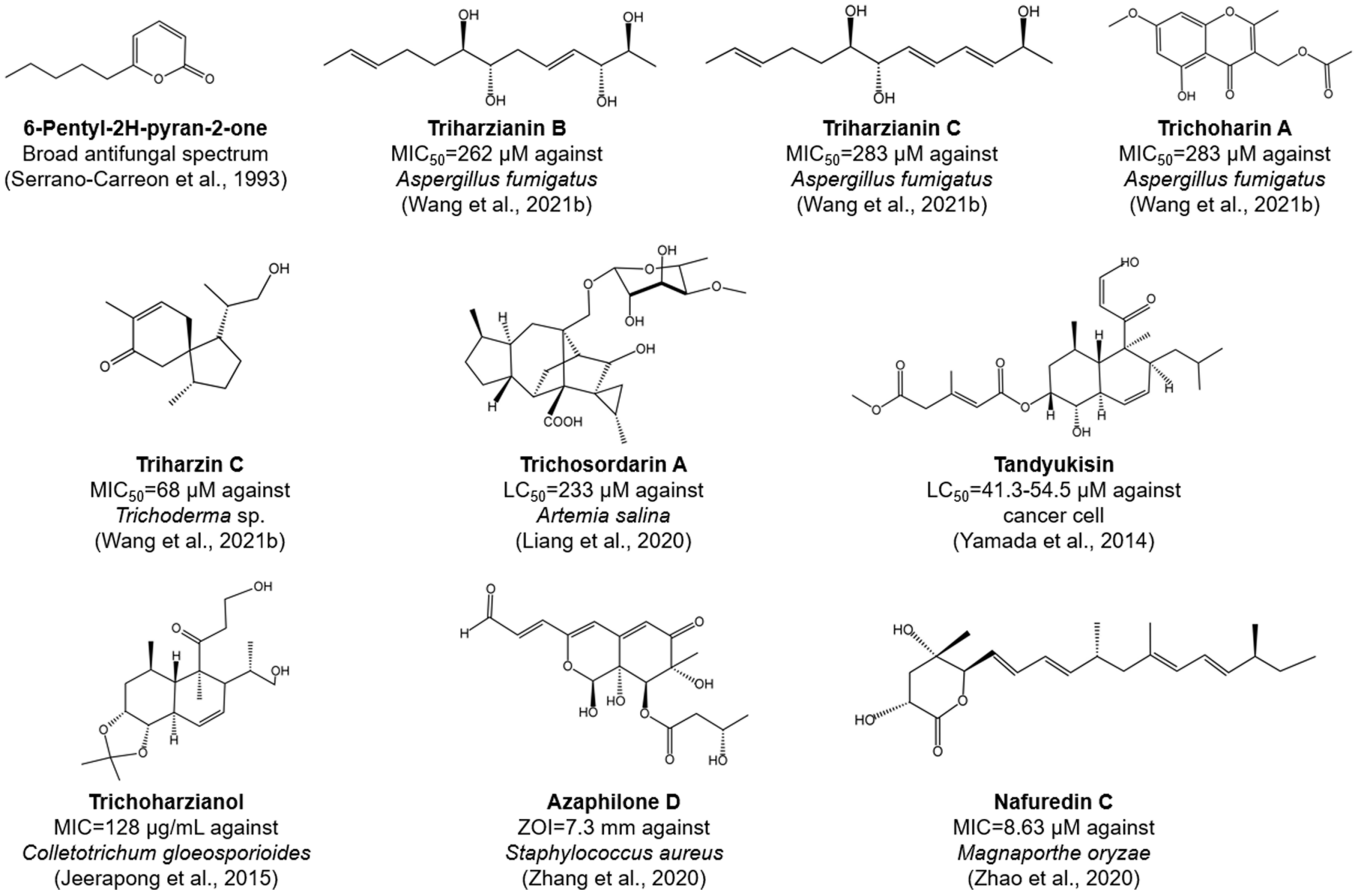
Chemical structures of selected bioactive compounds produced by *T. harzianum* species complex strains. MIC, minimum inhibitory concentration; LC, lethal concentration; ZOI, zone of inhibition.

The antibiosis activity of *T. harzianum* is generally mediated by the production of low-molecular-weight compounds, which can directly or indirectly inhibit the growth of pathogens. These include a variety of classes of compounds, such as peptides (McMullin et al., 2017; Kai et al., 2018; van Bohemen et al., 2021), polyketides (Zhao et al., 2020), and terpenes (Song et al., 2018; Figure 3). Various methods have been developed for the discovery of new metabolites with antimicrobial activity in *T. harzianum*. First, the one strain-many compounds (OSMAC) method was used to activate secondary metabolic gene clusters, which in turn altered their metabolic pathways to synthesize new metabolites (Yu et al., 2021). Using this method, eleven compounds were obtained from a *T. harzianum* strain, of which triharzianin B, triharzianin C, trichoharin A, triharzin C, 5-hydroxy-3-hydroxymethyl-2-methyl-7-methoxychromone, trichoacorenol B and harzianone exhibited antifungal activity against *Aspergillus fumigatus* and *Trichoderma* sp. (Wang X.-Y. et al., 2021). Second, the mutant strains of *T. harzianum* may produce new compounds. For example, several mutants obtained by UV mutagenesis exhibited increased *Fusarium*-inhibiting activity and produced two new compounds, including an isonitrile compound with broad antibiotic activity against fungi and bacteria (Graeme-Cook and Faull, 1991; Faull et al., 1994). Third, mining of new isolates of *T. harzianum* from soil, plant root systems, and rhizomes allowed for the identification of new chemical derivatives with inhibitory activities, such as α-pyrone and decalin derivatives (Nuansri et al., 2022), pentadecaibins (van Bohemen et al., 2021), azaphilone D and E (Zhang et al., 2020), harzianopyridone (Ahluwalia et al., 2015), tandyukisin (Yamada et al., 2014), trichosordarin A (Liang et al., 2020), trichoharzianol (Jeerapong et al., 2015), harzianic acid (Vinale et al., 2009), and nafuredin C (Zhao et al., 2020).

### 3.2. Elucidation and modification of the biosynthetic pathways of compounds

Understanding of the synthetic pathway is important for improving the production level and modifying the structures of natural products, which help to improve the inhibitory ability of *T. harzianum* against pathogens. Despite the extensive reports on bioactive compounds from *T. harzianum*, the biosynthetic pathways of most of these molecules remain unresolved so far.

#### 3.2.1. Lactones

Lactone compounds such as butenolides (e.g., harzianolide) and pyrones are commonly isolated from *T. harzianum.* Harzianolide could significantly promote tomato seedling growth and activate plant systemic resistance (Cai et al., 2013), and its biosynthesis pathway was shown to involve the rearrangements and decarboxylation of a heptaketide (Avent et al., 1992).

6-Pentyl-2H-pyran-2-one (6-PP) is an unsaturated volatile lactone with a coconut aroma, and is commonly detected in the secondary metabolites produced by *T. harzianum* and other *Trichoderma* species (Keswani et al., 2014; Vinale et al., 2014). 6-PP can inhibit the growth of a broad spectrum of fungal pathogens such as *Fusarium moniliforme* and *R. solani* (Scarselletti and Faull, 1994; El-Hasan et al., 2007). Furthermore, it can promote plant growth and induce plant defenses against pathogenic fungi (Garnica-Vergara et al., 2016; Lazazzara et al., 2021). Deciphering the 6-PP biosynthetic pathway is yet to be accomplished, and most of the clues from *T. atroviride* isotopic labeling experiments have suggested that the oxidation of linoleic acid by lipoxygenase might be a major step in the biosynthesis of 6-PP by *Trichoderma* (Serrano-Carreon et al., 1993). However, a gene deletion study showed that the single lipoxygenase-encoding gene *lox1* is dispensable for the production of 6-PP and for the antagonistic capacity of *T. atroviride* against the plant pathogen *B. cinerea* (Speckbacher et al., 2020). The authors proposed that the synthesis of 6-PP may involve the action of polyketide synthase. In addition, 6-PP can be degraded or converted into the intracellular microsomal fraction of *T. atroviride*, which decreases its concentration in culture (Flores et al., 2019).

#### 3.2.2. Sterols and terpenoids

**Figure 4:**
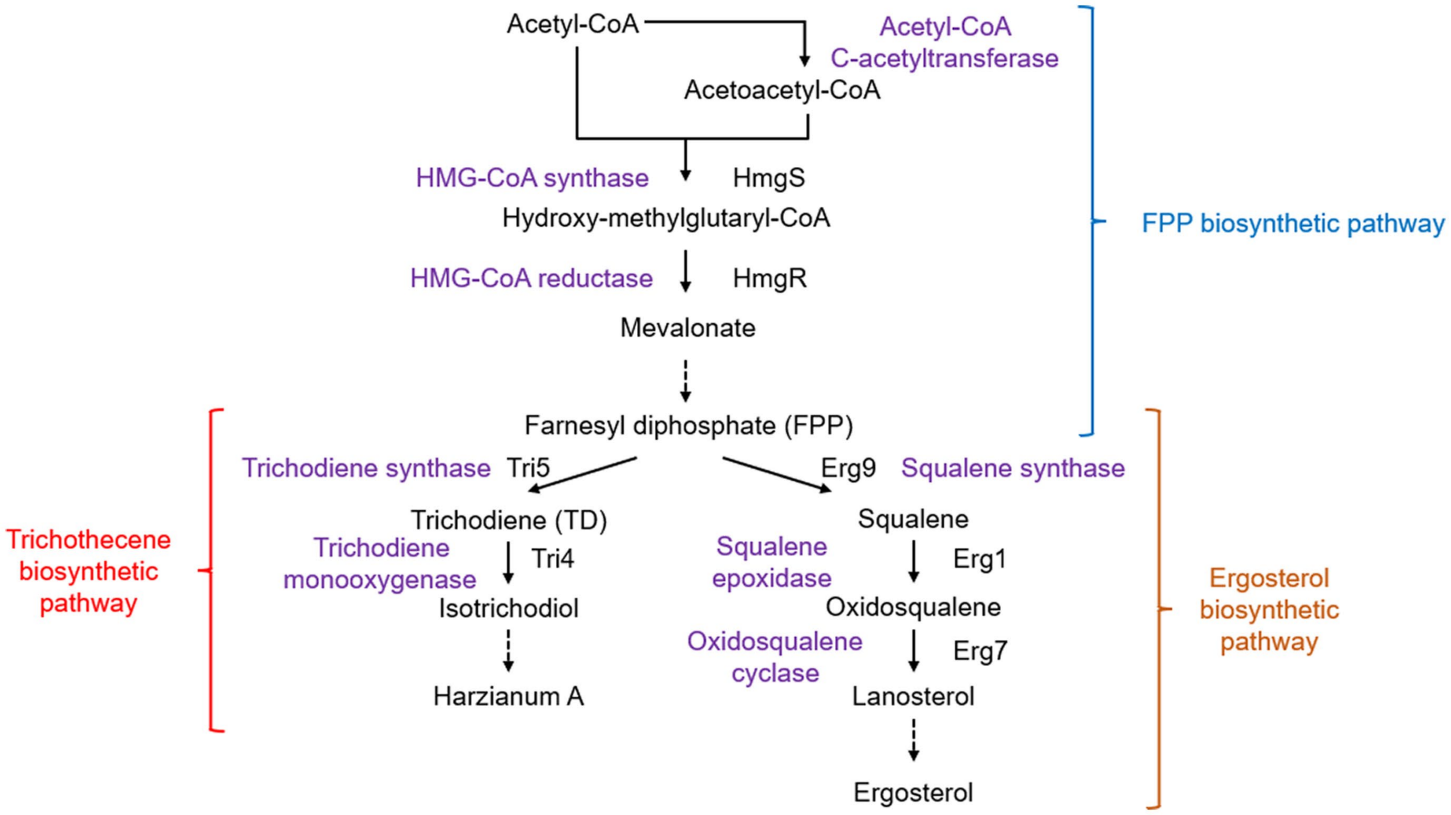
Biosynthetic pathways of sterol and trichothecenes. Solid arrows show direct chemical reactions, while dashed arrows represent a series of chemical reactions.

Ergosterol, a component of the fungal cell membrane, can upregulate the expression of plant defense-related genes and elicit responses through induction of the oxidative burst by inhibition of H^+^-ATPase activity on the plasma membrane (Rossard et al., 2010; Khoza et al., 2019). Hydroxy-methylglutaryl-CoA reductase (encoded by *hmgR*) is a rate-limiting enzyme involved in the synthesis of farnesyl diphosphate (FPP), an important intermediate in sterol synthesis (Figure 4). Partial silencing of the *hmgR* gene in *T. harzianum* led to a reduction in antifungal activity against the plant pathogens *R. solani* and *Fusarium oxysporum* and a 15.8-fold increase in the expression of *erg7* in the sterol pathway (Cardoza et al., 2007).

Silencing of the squalene epoxidase-encoding gene *erg1* led to lower ergosterol production and increased *erg7* expression (Cardoza et al., 2006b). In addition, silencing *erg1* was found to increase the production of squalene, which can induce the expression of tomato defense-related genes in a concentration-dependent manner. The ability of *T. harzianum* to colonize tomato roots has also been enhanced (Malmierca et al., 2015b). However, overexpression of *erg1*, although it did not show any effect on ergosterol levels, led to a substantial decrease in the amount of squalene and also reduced the priming ability of some defense-related genes in the salicylic acid and jasmonic acid pathways (Cardoza et al., 2014).

The synthesis of sesquiterpene compounds, including trichothecenes in fungi, also uses FPP as a precursor (McCormick et al., 2011). Many trichothecenes are fungal toxins with some showing good antifungal activity. *Harzianum* A, a non-phytotoxic trichothecene produced by *Trichoderma arundinaceum*, was found to have antagonistic activity against fungal pathogens and induce plant defense response genes (Malmierca et al., 2012). In trichothecene biosynthesis, the first step is to cyclize FPP to form trichodiene (TD) using trichodiene synthase encoded by *tri5* (Fekete et al., 1997). Although a *tri5* homologous gene has been isolated from *T. harzianum* ATCC 90237 (Gallo et al., 2004), this strain was later identified as *T. arundinaceum* (Degenkolb et al., 2008). Currently, *T. harzianum* is thought to be unable to synthesize trichothecenes. When *T. harzianum* was transformed with *tri5* from *T. arundinaceum*, the production of TD resulted in the upregulation of plant defense-related genes in tomatoes (Malmierca et al., 2015a). This TD-producing strain showed enhanced biocontrol activity against *F. graminearum* and reduced mycotoxin deoxynivalenol contamination (Taylor et al., 2022). Transgenic *T. harzianum* with both *tri5* and *tri4* produced 12,13-epoxytrichothec-9-ene and downregulated tomato genes involved in fungal root colonization and pathogen defense (Cardoza et al., 2015). These findings highlight the intricate interactions between host plants, fungal pathogens, and antagonists mediated by trichothecene compounds.

#### 3.2.3. Polyketides

Azaphilones as a family of polyketide-based secondary metabolites were isolated from the *T. harzianum* species complex. These compounds were shown to have antifungal, antiviral or radical scavenging activities (Vinale et al., 2006; Pang et al., 2020; Xie et al., 2022). The gene cluster for the biosynthesis of trigazaphilones in *T. guizhouense* has been identified (Pang et al., 2020). Another gene cluster *hac* is responsible for the biosynthesis of harzianic acid in *T. afroharzianum* and *T. guizhouense*, with two transcriptional activators identified to be involved in its regulation (Xie et al., 2021; Pang et al., 2022).

The products of many gene clusters containing polyketide synthase (PKS)-encoding genes in *T. harzianum* remain unknown. *In vitro* plate confrontation experiments against *S. sclerotiorum*, *R. solani*, and *F. oxysporum* revealed that the PKS-encoding genes *pksT-1* and *pksT-2* are differentially regulated in *T. harzianum* in response to fungal pathogens. The *pksT-2* knockout mutant showed a significant change in the color of the conidia, but the biocontrol activity of the mutant was not tested (Yao et al., 2016). Additionally, heterologous expression of a polyketide synthase-nonribosomal peptide synthetase gene cluster from *T. harzianum* in *Aspergillus nidulans* has led to the discovery of new tetronate compounds with potential antimicrobial activities (Zhu et al., 2021).

## 4. Enhancing the robustness of strains

In addition to the production of the biocontrol effectors mentioned above, it is also important to improve the resistance of *T. harzianum* to various stresses in practical applications. The survival characteristics of these strains may be significantly influenced by physical and chemical environmental factors such as pH, temperature, and fungicides in the soil (Lo et al., 1998). Therefore, the ecology of *T. harzianum* should be better understood to deploy biocontrol agents for disease control.

The synergistic application of fungicide and *T. harzianum* can reduce the amount of fungicide used while ensuring the same inhibition rate (Wang et al., 2019; Becker et al., 2021); however, this is based on a situation where *T. harzianum* shows resistance to fungicides. After exposure to UV light, mutant strains obtained by screening on specific plates supplemented with fungicides showed cross-resistance to prochloraz and bromuconazole (Figueras-Roca et al., 1996) or to benomyl and thiabendazole (Hatvani et al., 2006). *Thmfs1*, a major facilitator superfamily transporter gene, is partially responsible for trichodermin secretion in *T. harzianum*. A strain overexpressing *Thmfs1* displayed increased resistance to a wide range of antimicrobial compounds (Liu et al., 2012; Table 3). In addition to chemical fungicides, the tolerance to metabolites secreted by pathogenic fungi should be taken into the consideration. For example, the metabolite fusaric acid produced by *F. oxysporum* inhibits the growth of *T. harzianum*. A UV-C mutant was not only more tolerant to fusaric acid but also more effective against *Fusarium* wilt in tomatoes than the wild-type (Marzano et al., 2013).

**Table 3:** Examples of *T. harzianum* strain improvement for higher resistance to fungicides.

Fungicide	Method of strain development	Increase in resistance^a^	Reference
Bromuconazole	Exposure to UV radiation	MIC from 25 to >125 μg/ml	Figueras-Roca et al. (1996)
Prochloraz	Exposure to UV light and then selecting by colony morphology on prochloraz amended media	MIC from 1 to >12.5 μg/ml	Figueras-Roca et al. (1996)
Methyl benzimidazole-2-yl carbamate (MBC)	Exposure to UV-A light and then selecting on MBC amended media	Increased tolerance from 0.4 to 100 μg/ml	Hatvani et al. (2006)
Phosetyl aluminum	Exposure to UV light	EC_50_ = 1,043.20 μg/ml, a 13.76-fold increase over the parental strain	Besoain et al. (2007), Herrera et al. (2012)
Potassium phosphite	Treatment with N-methyl-N-nitro-N-nitrosoguanidine and then selecting on amended media	EC_50_ = 12,503.10 μg/ml, a 288.09-fold increase over the parental strain	Perez et al. (2007), Herrera et al. (2012)
Tebuconazole and the other eight compounds	Overexpression of *Thmfs1* using CaMV35S promoter	4- to 12-fold increase of MIC	Liu et al. (2012)

^a^EC, effective concentration; MIC, minimal inhibitory concentration.

Besides the resistance to antifungal chemicals, the tolerance to other abiotic stresses needs to be taken into account when applying *T. harzianum* in specific environments. The response to heat stress is a highly conserved system by inducing the synthesis of heat-shock proteins (Lindquist and Craig, 1988). When *T. harzianum* conidia were heat-shocked at 45°C for 2 h, the *hsp70*-overexpressed strains showed better growth than the wild-type under various oxidative, osmotic, and salt stresses (Montero-Barrientos et al., 2008). Transformants with the superoxide dismutase (SOD)-encoding gene showed a significantly higher resistance to heat and salt stress. Although the wild-type strain could not grow at 40°C or in the presence of 2 mol/l NaCl, the *sod* transformant maintained its inhibitory activity against *S. sclerotiorum* under these conditions (Yang et al., 2010). In addition, hydrophobins play important roles in the resistance of *Trichoderma* spores to several kinds of abiotic stresses (e.g., UV radiation), and the perturbation of hydrophobin-encoding genes can result in species-specific changes of phenotypes (Cai et al., 2020).

Mycoviruses are widely observed among fungal species, some of which are harmful to their hosts. Recently, *T. harzianum* hypovirus 1 (ThHV1) was identified in a *T. harzianum* isolate, and strains carrying both ThHV1 and its defective RNA were found to have a decreased mycoparasitism ability (You et al., 2019). Therefore, the viruses in *T. harzianum* may also be related to their stable performance.

## 5. Modulation of the gene regulatory system

The synthesis of protein and chemical effectors, as well as the responses to environmental stresses, are tightly regulated in *Trichoderma* species for biocontrol. In eukaryotes, gene regulatory systems respond to external signals and typically undergo multiple signal transitions to regulate downstream gene expression. Modification of the gene regulatory system can often alter the expression levels of multiple genes simultaneously, making it an efficient strategy for strain engineering (Pang et al., 2022).

### 5.1. Signaling pathways

The sensing of pathogenic fungi and the consequent responses in *Trichoderma* involve the combinatorial action of different signaling pathways (Howell, 2003; Zeilinger and Omann, 2007). Several classical signal transduction pathways in fungi have been linked to their ability to combat phytopathogens in *Trichoderma* spp., including G protein signaling, mitogen-activated protein kinase (MAPK) cascades, and cAMP pathways (Mendoza-Mendoza et al., 2003; Omann and Zeilinger, 2010).

Heterotrimeric G-protein complexes consist of α, β, and γ subunits, and most filamentous fungi have three Gα subunits: Gα1, Gα2, and Gα3. Knockout of the *Thga1* gene, which encodes the GαI protein, led to reduced growth rate, decreased 6-PP and chitinase production, and complete loss of the capacity to overgrow and lyse *R. solani*, *B. cinerea*, and *S. sclerotiorum* during *in vitro* plate confrontation (Sun et al., 2016). Knockout of another Gα-encoding gene, *Thga3*, results in an 80% reduction in hydrophobin expression and a 23% reduction in chitinase activity (Ding et al., 2018, 2020). Despite the demonstrated role of G proteins, the function of G protein-coupled receptors (GPCRs) has not yet been studied in *T. harzianum*. In *T. atroviride*, silencing of Gpr1, a cAMP-receptor-like family GPCR, results in the loss of capacity to activate the expression of chitinase and protease genes and to attach to host hyphae (Omann et al., 2012).

Highly conserved MAPK cascades play a crucial role in the transmission of extracellular and intracellular signals in fungi by controlling transcription factors through a phosphorylation cascade (Martínez-Soto and Ruiz-Herrera, 2017). *hog1* is a homolog of the MAPK-encoding gene *HOG1*, controlling the hyperosmotic stress response in *Saccharomyces cerevisiae*. A mutant strain containing hyperactive point-mutated *hog1* and another with *hog1* silencing was constructed in *T. harzianum*. Both mutant strains showed strongly reduced antagonistic activity against the plant pathogens *Phoma betae* and *Colletotrichum acutatum* (Delgado-Jarana et al., 2006).

### 5.2. Transcriptional regulatory system

**Figure 5:**
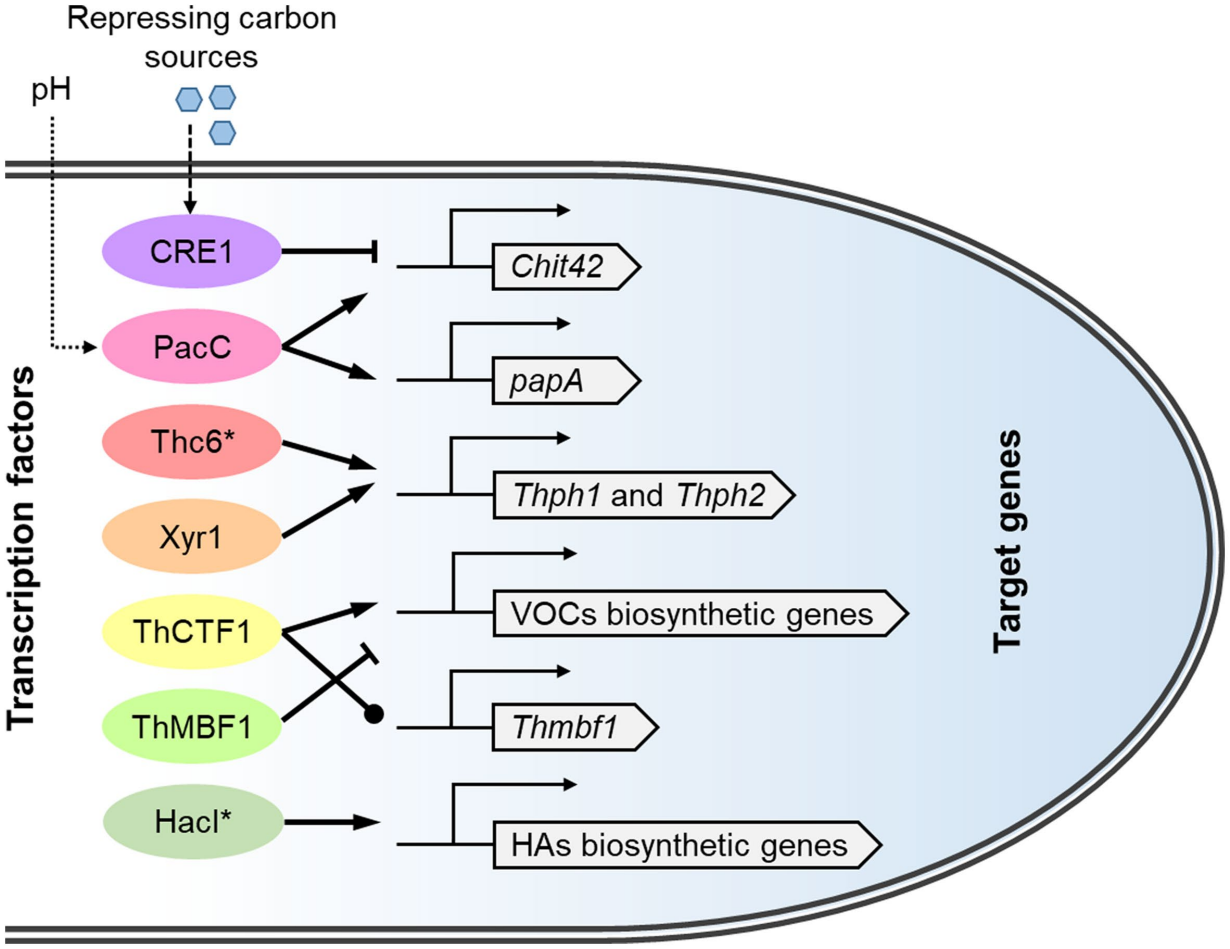
The transcriptional regulatory system of biocontrol-related genes in *T. harzianum* species complex. The transcription factors respond to upstream signals and regulate the expression of target genes involved in biocontrol. The corresponding protein IDs in *T. afroharzianum* T-22 (https://mycocosm.jgi.doe.gov/TriharT22_1/TriharT22_1.home.html) are: CRE1, 298,239; PacC, 516,100; Thc6, 503,211; Xyr1, 455,911; ThCTF1, 348,498; ThMBF1, 493,750; HacI, 207,786; *chit42*, 6,140; *papA*, 315,275; *Thph1*, 627,343; *Thph2*, 555,537. VOCs, Volatile organic compounds; HAs, harzianic acids. Transcription factors reported to be engineering targets for strain improvement are marked with asterisks.

The significant changes in the transcriptome of *T. harzianum* during interactions with fungal pathogens involve the action of a set of transcription factors (Figure 5). CRE1, a conserved carbon catabolite repressor in fungi, is the first demonstrated transcription factor in the biocontrol with *T. harzianum*. Before contact with the plant pathogen, CRE1 can bind to two single sites in the promoter of chitinase gene *chit42* to inactivate its expression. Confrontation with *B. cinerea* relieved the binding of Cre1 to the *chit42* promoter (Lorito et al., 1996). In contrast, the expression of *chit42* is triggered by soluble chitooligosaccharides which can be produced by constitutive chitinolytic enzymes (Zeilinger et al., 1999). In addition, a BrlA-like binding motif in the *chit42* promoter was found to be related to the regulation of its expression in *T. atroviride* (Brunner et al., 2003).

The zinc cluster family transcription factor Thc6 is involved in the induction of systemic plant resistance by *T. harzianum*. Overexpression mutants of Thc6 could activate the expression of the jasmonic acid pathway genes and reduce the disease index of maize treated with *Curvularia lunata* (Fan et al., 2015). As a homolog of the cellulase transactivator ACE2 in *T. reesei*, Thc6 can bind to the promoters of cellulase genes *Thph1* and *Thph2*. Knockout mutants of these two genes resulted in the loss of the ability to activate the expression of immune defense-related genes in plants (Saravanakumar et al., 2016, 2018).

Another zinc cluster transcription factor, ThCTF1, is involved in regulating the synthesis of 6-PP in *T. harzianum*. The *Thctf1* deletion mutant did not produce two secondary metabolites derived from 6-PP and showed reduced antimicrobial capacity (Rubio et al., 2009). Through suppression subtractive hybridization between the wild-type strain T34 and a *Thctf1*-null mutant, a helix-turn-helix family regulator, ThMBF1, was identified to be differentially expressed. Overexpression of *Thmbf1* exacerbated the incidence of fungal diseases in tomato plants, suggesting that this gene has a negative role in the biocontrol process (Rubio et al., 2017).

The transcription factor PacC/Rim101 plays an important role in adaptation to ambient pH in fungi (Denison, 2000; Franco-Frías et al., 2014). Pac1/ThPacC (homologous to PacC/Rim101) regulates many genes involved in *T. harzianum* antagonism, such as *chit42* and protease *papA*. The silencing of *pac1* seems to promote the production of certain metabolites that inhibit some plant pathogenic fungi, but it negatively affects the parasitic capacity of *T. harzianum* (Moreno-Mateos et al., 2007). Another study revealed that the *ThpacC* knockout strain did not produce the antifungal molecules homodimericin A and 8-epi-homodimericin A and showed reduced inhibition against *S. sclerotiorum* (Wu et al., 2021). However, neither constitutive activation nor overexpression of Pac1/ThPacC increased biocontrol ability in the above two studies.

## 6. Future perspectives

### 6.1. Further discovery and characterization of the molecules related to biocontrol

The biocontrol capacity of *T. harzianum* involves complex interactions between the pathogens and plants. To date, the molecular mechanisms underlying the action of many effector proteins and compounds against phytopathogens have not been fully elucidated. The activities of these effector molecules and their combinatorial effects on different types of pathogens need to be investigated in detail to guide strain engineering. In particular, attention should be paid to the effects of the molecules or strains on the defense response and growth of plants, in addition to the results of traditional plate confrontation experiments.

The sequencing and annotation of the *T. hazianum* genome enabled the discovery of effector proteins and metabolites connected to biocontrol activity on a large scale (Rush et al., 2021). According to annotations from the JGI MycoCosm portal,^1^ there are approximately 60 secondary metabolic gene clusters in most sequenced strains in the *T. harzianum* species complex. For silent biosynthetic gene clusters, their products are expected to be identified and increased for production using molecular biology tools, such as promoter exchange, overexpression of pathway-specific transcription factors, and heterologous expression (Brakhage and Schroeckh, 2011). The genome-driven approach has been used to mine bioactive natural products from *T. harzianum*, which resulted in the discovery of several unique compounds and widened the knowledge of their biosynthetic pathways (Chen et al., 2019; Zhu et al., 2021).

Transcriptomic and secretomic analyses have suggested that hundreds of genes in *T. harzianum* are significantly differentially expressed during interaction with fungal pathogens (Vieira et al., 2013; Steindorff et al., 2014; Ramada et al., 2016). Systematic investigation of the functions of these genes can provide more targets for engineering strains with enhanced biocontrol capacities. For example, aquaglyceroporin, which facilitates the transport of water and solutes across the membrane, was found to be significantly upregulated in *T. harzianum* during its interaction with *F. solani*. A transformant overexpressing its encoding gene was capable of significantly reducing *Fusarium* sp. growth compared to the wild-type (Vieira et al., 2018).

Additionally, although the defective RNA of ThHV1 decreases the pathogen-inhibitory ability of *T. harzianum*, some other viruses enhance mycoparasitic ability by regulating the activity of cell wall-degrading enzymes. Compared to ThPV1-cured strains, β-1,3-glucanase activity and the ability to combat *P. ostreatus* and *R. solani* were increased in ThPV1-containing strains (Chun et al., 2018). In the future, dsRNA in *T. harzianum* strains can be mined from their genomes to identify beneficial viruses for improving their biocontrol abilities. Nevertheless, the effects of virus-infected *T. harzianum* strains on the physiological characteristics of plants and the plant root microbiome need to be studied.

### 6.2. Deeper understanding of the gene regulatory system

Overexpression, mutagenesis, and domain-swapping strategies have been successfully used to engineer regulatory proteins in filamentous fungi (e.g., *T. reesei*) to improve the production of plant biomass-degrading enzymes (Liu and Qu, 2021; Zhao et al., 2022). However, understanding of the roles of regulatory proteins in biocontrol is still limited. Through the construction of gene disruption mutants, MAPKs, adenylate cyclase, protein kinase A, and GTPase activators have been linked to the inhibition of pathogens and production of secondary metabolites in *Trichoderma* species (Mukherjee et al., 2003; Hinterdobler et al., 2019; Segreto et al., 2021). These signaling proteins might have similar functions in *T. harzianum* and need to be studied and tested as potential targets for strain engineering in the future.

Despite the studies summarized in Figure 5, knowledge of the transcriptional regulation of biocontrol-related genes in *T. harzianum* is fragmented. Transcriptional activator(s) binding to the promoters of chitinase-encoding genes have yet to be identified (Lorito et al., 1996). Overexpression or improvement in the activity of such activators is expected to increase the expression of a set of fungal cell wall-degrading enzymes. In *T. atroviride*, the xylanase transcriptional regulator XYR1 positively regulates the expression of lignocellulolytic enzyme genes and activation of plant defense responses (Reithner et al., 2014). Overexpression of *xyr1* has been shown to increase the production of cellulases and xylanases in *T. harzianum* (da Silva Delabona et al., 2017), but its effect on biocontrol ability needs to be studied.

In addition to transcriptional factors, proteins that regulate chromatin structure can significantly affect the expression levels of targeted genes. The *lae1* (encoding putative methyltransferase) and *tgf-1* (encoding histone acetyltransferase) genes were proven to be related to mycoparasitism in *T. atroviride* (Karimi Aghcheh et al., 2013; Gómez-Rodríguez et al., 2018). Overexpression of *lae1* in *T. harzianum* results in a significant increase in cellulolytic gene expression (Delabona et al., 2020), and its function in secondary metabolite synthesis and biocontrol is worth investigating.

### 6.3. Strain engineering and design in the synthetic biology era

Based on the understanding of the molecular mechanisms for biocontrol, systems metabolic engineering strategies could be employed to construct *T. harzianum* strains with increased pathogen-inhibiting capacity and enhanced robustness (Ko et al., 2020). For the identified effector proteins and compounds, cutting-edge technologies for protein engineering and combinatorial biosynthesis are expected to be used to modify their structures for higher activities toward pathogens (Staunton and Wilkinson, 2001). The introduction of heterologous genes related to biocontrol is another approach to improve the ability of *T. harzianum* to combat pathogens. An insect-specific neurotoxin gene from the scorpion *Androctonus australis* was heterologously expressed in *Metarhizium anisopliae*, which significantly increased its ability to kill pest insects (Wang and St Leger, 2007). Similarly, heterologous genes (e.g., peptaibol synthetic gene clusters from other *Trichoderma* species) may be introduced into *T. harzianum* to expand the range of its action. The safety of the engineered strains, however, should be carefully evaluated, and the transfer of transgenic genes should be well controlled (Stirling and Silver, 2020).

The multiplex genetic engineering of strains requires the development of highly efficient gene manipulation tools. Traditionally, polyethylene glycol-mediated and *Agrobacterium*-mediated transformation methods have been used to construct mutants in *T. harzianum* (Cai et al., 2021), which allowed gene overexpression and targeted genetic recombination (e.g., gene knock-out). New methods for strain engineering, for example, CRISPR/Cas9-based genome editing, have offered straightforward platforms to carry out multiplex genetic modifications in filamentous fungi (Kun et al., 2019; Wang Q. et al., 2021). The first application of CRISPR/Cas9-based genome editing in *Trichoderma* was reported in *T. reesei* (Liu et al., 2015). Through recycling of selection marker genes, consecutive rounds of gene deletion were achieved in *T. reesei* (Chai et al., 2022). Recently, this technique was used in *T. harzianum* to inactivate the *pyr4* gene to construct an uracil-deficient strain (Vieira et al., 2021). The genome editing methods also have the advantage of being easy to achieve genetic modifications without introduce foreign DNA, which can overcome some restrictions on the use of GMOs. For heterologous expression of biosynthetic gene clusters, the assembly of large DNA fragments has been reported based on homologous recombination in yeast or directly in filamentous fungus (Chiang et al., 2021). These methods are expected to aid in the systematic genetic modification of *T. harzianum* for the development of next-generation biocontrol agents.

To be used under field conditions, the strains in biocontrol agents are required to be genetically stable and eco-friendly. Exogenous DNA is usually integrated to the chromosome to ensure stability in strain engineering of *Trichoderma* (Cardoza et al., 2006a; Yang L. et al., 2011). So far, the only element reported for autonomous replication of plasmids in *Trichoderma* is AMA1 from *A. nidulans* (Kubodera et al., 2002). Such plasmids are easy to lose and not suitable for the construction of improved strains for practical application. On the other hand, the use of antibiotic-resistance genes as selectable markers in strain engineering could pose a threat to environment and public health. Therefore, it is better to use selection markers other than antibiotic-resistant genes (e.g., auxotrophic markers) or to remove antibiotic-resistance genes in the final strains (Zhao et al., 2016). With the use of advanced genetic manipulation technologies and well-implemented risk assessments, engineered biocontrol strains have the potential to step out of laboratories to increase agricultural production in the near future.

## Author contributions

ZX and GL drafted the manuscript. GL, LG, and WL revised the manuscript. All authors collected literature information and discussed about the organization of the manuscript, read, and approved the final manuscript.

## Funding

This work was supported by National Natural Science Foundation of China (32100088), Shandong Natural Science Foundation (ZR2021QC073), Young Scholars Program of Shandong University (YSPSDU to GL), and Science and Technology Project of Shanghai Tobacco Group Beijing Cigarette Factory Co., Ltd. (TP2021-T2). The authors declare that the funder Shanghai Tobacco Group Beijing Cigarette Factory Co., Ltd., had the following involvement in the study: collection of literature and the writing of this article.

## Conflict of interest

WL was employed by Shanghai Tobacco Group Beijing Cigarette Factory Co., Ltd.

The remaining authors declare that the research was conducted in the absence of any commercial or financial relationships that could be construed as a potential conflict of interest.

## Publisher’s note

All claims expressed in this article are solely those of the authors and do not necessarily represent those of their affiliated organizations, or those of the publisher, the editors and the reviewers. Any product that may be evaluated in this article, or claim that may be made by its manufacturer, is not guaranteed or endorsed by the publisher.

## Footnotes

^1^ https://mycocosm.jgi.doe.gov/mycocosm/home
